# Contextualised peak periods of play in English Premier League matches

**DOI:** 10.5114/biolsport.2022.112083

**Published:** 2021-12-30

**Authors:** Wonwoo Ju, Dominic Doran, Richard Hawkins, Antonio Gómez-Díaz, Andres Martin-Garcia, Jack D Ade, Andy Laws, Mark Evans, Paul S Bradley

**Affiliations:** 1 The Research Institution for Sport and Exercise Sciences at Liverpool John Moores University, Liverpool, England, UK; 2 Football Medicine and Science Department at Manchester United Football Club, Manchester, UK; 3 The Poland National Football Team, Polish Football Association, Warsaw, Poland; 4 FC Barcelona Sports Performance Department, Barcelona, Spain; 5 Liverpool FC Academy, Liverpool Football Club, Liverpool, UK; 6 Department of Computer Science, Liverpool John Moores University, Liverpool, UK; 7 Football Science Consultant, UK

**Keywords:** Match performance, Peak periods, Transient decrements, Physical-tactical data, Football

## Abstract

The present study aimed to determine the physical-tactical trends of elite players/teams during peak 1-, 3- and 5-min periods of match-play. A total of 50 English Premier League matches (n = 583 player observations) were analysed by coding the players’ physical-tactical activities through the synchronisation of tracking data and video. The contextualised data showed that during the peak periods (i.e., the most demanding passage of play), players/teams covered the largest distances for ‘Recovery Run’ (28–37%) out of possession and ‘Support Play’ (9–13%) in possession. In the following periods, players covered less high-intensity distance versus the average with a more pronounced decline in the next 1-min period than longer duration 3- and 5-min periods (48% vs ~25–30%, ES: 0.4–0.5, *P* < 0.01); team data showed similar trends with different relative patterns (31% vs 17–30%, ES: 0.5–0.8, *P* < 0.01). After peak periods, players/teams performed 20–53% less high-intensity distances for ‘Covering’ and ‘Recovery Run’ (ES: 0.2–0.7, *P* < 0.01) out of possession. However, players covered 28–91% less distance for ‘Run with Ball’ (ES: 0.1–0.5, *P* < 0.05) when in possession. Some physical-tactical actions exhibited inconsistency in different time durations of the next periods; however, these physical-tactical data were position-specific. This may signify that each position has certain physical-tactical actions to execute even after the peak periods, especially when they are tactically required to do so. As the data demonstrates unique physical-tactical trends of players/teams during the peak and next periods of play, this could help practitioners prescribe position- and player-specific drills, and better understand transient decrements in high-intensity running after intense passages of play.

## INTRODUCTION

Time-motion analysis has been widely used for profiling the match performance of elite players [1–4]. Practically, the activity profiles derived from match-play are used for designing training drills [5, 6]. However, previous studies have mainly analysed the average physical demands [3, 7, 8], which underestimates locomotive demands of players [9]. Hence, greater attention has been paid to the physical demands during the peak period (i.e., the most intense period of a match) [9–13]. Although peak performance data have been practically used as a benchmark to devise football-specific drills [6, 14], issues exist when attempting to directly translate these into specific drills as the context of play is completely omitted from any of the studies that have quantified match-play peak periods [15, 16]. Thus, tactical context should be fused with physical metrics to help coaches prescribe specific drills that mimic these intensified periods of matches more effectively.

Football (soccer) is a team sport where player’s physical and tactical actions are influenced by both opponent and teammate’s actions [15]. Nevertheless, previous studies included only player performances to understand individual patterns rather than actual team trends [11, 13, 17]. Quantifying individual players limits our understanding of a team’s collective performance during match-play. Thus, analysing team trends during intensified periods of play could provide new insights and help with team-based drill prescriptions [5, 14]. No research, to the best of our knowledge, has attempted to observe the peak physical demands for team performances. Therefore, analysing team’s collective physical-tactical performances could add insights into how teams collectively perform physical-tactical actions during intensified periods of play together with individual player data.

Several studies have investigated not only peak periods of play over different time durations (e.g., 1-, 3-, 5-min) but the 5-min periods after intense periods during match-play to examine transient decrements in high-intensity running compared to the match average [1, 3, 18]. The immediate declines in physical performance during the next 5-min periods have been ascribed to fatigue induced by the activities during peak periods. Although it is highly complex, there seems to be several contributing factors that cause fatigue (e.g., muscle acidosis and reduced muscle creatine phosphate) after intense periods [19]. However, temporary declines in high-intensity running are not necessarily linked to fatigue but could be due to pacing strategies/tactical alterations [20] and/or less playing opportunities [21]. To potentially understand ‘HOW’ players/teams alter their tactical behaviour during the phases that follow intense periods of match-play, amalgamating physical and tactical performance data could be a solution [22].

Previous research examining transient decrements in high-intensity running in the next period after the most demanding passage of a match had several limitations [1–3, 18]. Most studies used a predefined period (e.g., 0–5, 5–10 min etc.), which can under or over-estimate the physical demands during the peak and the following periods, respectively [23]. Thus, it is more advisable to use a rolling average technique (distance covered from every time point) to provide a more precise estimation of physical demands during such periods [11, 13, 17]. Moreover, studies investigating transient decrements used only a 5-min interval for the next period after the most intense period of play, which could omit brief changes immediately after intense actions [2, 18]. Hence, using shorter durations of the next period after the most intense passage of play may be more advantageous to understand short-term fluctuations. Therefore, the present study aimed to determine the physical-tactical profiles of elite players/teams during peak 1-, 3- and 5-min periods of high-intensity running and the subsequent periods of each time duration during match-play.

## MATERIALS AND METHODS

### Match Analysis and Player/Team Data

Match physical-tactical data were collated from the 2018–19 English Premier League season using an integrated approach and a new filter established for this research. Players’ behaviours were captured by cameras situated at roof level during matches and their physical-tactical actions were manually coded using the integrated approach. The validity and reliability of the integrated approach and the novel filter used were previously verified by Ju et al. [22]. The validity of the integrated approach demonstrated a strong agreement between the responses of both UEFA qualified coaches and performance analysts versus the gold standard responses (~92%), and its inter- and intra-observer reliability was a strong (κ = 0.81) to almost perfect (κ = 0.94), respectively. The novel filter isolated high-intensity activities reaching speeds > 19.8 km · h^−1^ for a minimal dwell time of 1 s [24].

The researcher completed 350 hours of coding to analyse 50 competitive matches and 1,265 player observations within 20 different teams. For individual player’s analysis, only outfield players who had completed the entire match in the same position were included (583 player observations). This consisted of 179 Central Defensive players (CDP), 147 Wide Defensive players (WDP), 167 Central Midfield players (CMP), 54 Wide Offensive players (WOP), and 36 Central Offensive players (COP). However, all of the player’s contextualised performances for each match were summarised to analyse team performances (players who were subbed in or out were included; 100 match observations). All data were analysed for the duration of each half, including stoppage time. Prior to analysis, all original data were anonymised to ensure confidentiality. Ethical approval was granted by Liverpool John Moores University (LJMU) research ethics committee.

### Match Control and Data Balance

To improve the scientific rigor of the research design, matches were arbitrarily selected while simultaneously controlling situational factors (e.g., team/opponent standards, locations, and seasonal phases) [25]. Thus, the number of matches for each parameter was initially balanced. Matches were excluded if goal differential was > 3 and a player dismissal occurred since these influence match running performances [20, 26].

### The Integrated Approach of Match Performance

High-intensity actions isolated by the novel filter were synchronised with video footage of all players throughout matches to code the tactical purpose of each action (Table 1). All coding occurred using QuickTime Player (Apple Inc, Cupertino, California) to watch video and then categorise tactical actions.

**TABLE 1:** The descriptions of the variables within the integrated approach.

Variables	Description
**In Possession**	
Push up Pitch	Player moves up the pitch to play offside and/or to squeeze to a higher line.
Break into Box	Player enters the opposition’s penalty box to receive the ball (typically receive ball from a cross – ball in front and wide).
Run in Behind/Penetrate	Player attacks space behind, overtakes and/or unbalances the opposition defence (typically ball is behind).
Over/Underlap	Player runs from behind to in front of the player on the ball or receiving the ball.
Run with Ball	Player moves with the ball either dribbling with small touches or running at speed with fewer ball touches.
Move to Receive/Exploit Space	Player moves to receive a pass from a teammate or to create/exploit space (typically come short or move wide to receive ball).
Support Play	Player supports from behind/level by trying to engage in offensive/transition play (typically during fast transitions).
**Out of Possession**	
Interception	Player cuts out pass.
Recovery Run	Player runs back towards their own goal to be goal side when out of position.
Covering	Player moves to cover space or an opposition player while remaining goal side.
Close Down/Press	Player runs directly towards opposition player on or receiving the ball, or towards space or players not on/receiving the ball.
**Unclassifiable**	
Other	All other variables that could not be categorised by the above.

The coding process was as follows: high-intensity actions with one tactical action were classified as a single action with dual tactical actions being coded as a hybrid action. High-intensity actions with more than three tactical actions were classified as ‘Other’. If the high-intensity action consisted of 70–90% of the primary and 10–30% of the secondary action, it was classified as a hybrid action. But if it was made up of 50–60% of the primary and 40–50% of the secondary action, then it was classified as ‘Other’. As hybrid actions are a combination of the primary and secondary actions [27], single action events and the primary tactical movements of the hybrid actions were combined to simplify data outputs.

### Physical-Tactical Performance for The Peak Period

Using a rolling average method, the peak periods of high-intensity running during matches for three different time durations (1-, 3-, and 5-min) were determined [11]. These durations were selected firstly, to facilitate a more detailed examination of temporal changes than using only a 5-min time interval [18, 28], and secondly to correspond with the typical duration of training drills [17]. The next period after the peak of each time duration was used to evaluate physical performance decrements by comparing them with the average of the match that the period occurred in [3]. In addition, this allowed exploration of how players/teams changed their tactical behaviour after intense periods of play. The mean distances of matches were calculated by averaging distances covered in all of the 1-, 3-, and 5-min periods excluding stoppage time [3, 18]. Nevertheless, when the amount of the remaining time during the following intense period was not equivalent to the peak period, the related data were removed from analysis.

### Statistical Analyses

Data are expressed as the mean ± standard deviation. All statistical analyses were conducted using IBM SPSS Statistics for Mac OS X, version 26 (IBM Corp., Armonk, N.Y., USA). Data normality was verified by Shapiro-Wilk and Kolmogorov-Smirnov tests. Differences between 1-, 3-, or 5-min periods within a game were determined using one-way analysis of variance (ANOVA) with repeated measures. Differences between playing positions were determined using one-way ANOVA. In the event of a significant difference, Bonferroni post hoc tests were used to identify any localised effects. Statistical significance was set at *P* < 0.05. Effect size (ES) for the meaningfulness of the difference was determined as follows: trivial (≤ 0.2), small (> 0.2–0.6), moderate (> 0.6–1.2), large (> 1.2–2.0) and very large (> 2.0–4.0) [29].

## RESULTS

### Contextualised Peak Periods – Individual Trends

During the peak 1-, 3- and 5-min periods, the players covered 28–34% and 22–25% of the high-intensity distance (67 ± 19 m, 92 ± 28 m, and 113 ± 36 m, respectively) performing ‘Recovery Run’ and ‘Covering’ actions, respectively. In possession, the largest proportion of the high intensity distance (11%) was covered for ‘Support Play’. In the next 1-, 3- and 5-min periods, the players experienced a deficit of 48%, 30%, and 25%, respectively, in high-intensity distance compared to the match average (ES: 0.4–0.5, *P* < 0.01). Out of possession the players covered 22–44%, 34–43%, and 27–45% less high intensity distance for ‘Covering’ (ES: 0.2–0.3, *P* < 0.01), ‘Recovery Run’ (ES: 0.2–0.3, *P* < 0.01), and ‘Close Down/Press’ (ES: 0.1–0.2, *P* < 0.05), respectively, compared to the match average whilst also performing 28–91% less ‘Run with Ball’ distance when in possession (ES: 0.1–0.5, *P* < 0.05). Table 2 illustrates the average distance per action with the number of actions across various positions during the peak periods.

**TABLE 2:** Average distance (m) per physical-tactical action and average number of actions during the peak periods per game across positions.

Position	Time	HIR Distance (m/min)	Average No. Actions [min-max]	In Possession						Out of Possession			Overall
				SP	MTR/ES	OVL/UDL	RWB	RIB/PEN	BIB	CD/PRE	COV	RR	
CDP	1 min	55 ± 17^*^	2 ± 1 [1–4]	73 (1)	29 ± 18 (6)	0 (0)	25 ± 13 (19)	0 (0)	18 ± 11 (5)	15 ± 7 (7)	30 ± 15 (119)	48 ± 17 (95)	38 ± 17 (273)
	3 min	24 ± 7^*^	3 ± 1 [1–5]	73 (1)	26 ± 13 (9)	0 (0)	22 ± 11 (29)	0 (0)	25 ± 16 (3)	17 ± 7 (15)	24 ± 10 (209)	40 ± 17 (119)	30 ± 12 (417)
	5 min	17 ± 5^*^	3 ± 1 [1–7]	0 (0)	23 ± 13 (12)	46 (1)	20 ± 10 (25)	0 (0)	18 ± 11 (5)	17 ± 7 (15)	22 ± 8 (272)	37 ± 17 (141)	27 ± 10 (518)
WDP	1 min	76 ± 18^#^	2 ± 1 [1–4]	38 ± 18 (43)	30 ± 17 (22)	47 ± 18 (18)	28 ± 13 (17)	21 ± 9 (10)	23 ± 7 (4)	16 ± 6 (20)	30 ± 14 (72)	41 ± 17 (92)	36 ± 12 (327)
	3 min	35 ± 8^#^	4 ± 1 [2–7]	31 ± 17 (65)	29 ± 14 (25)	34 ± 16 (29)	23 ± 14 (25)	23 ± 12 (18)	23 ± 8 (5)	15 ± 5 (34)	26 ± 12 (125)	36 ± 16 (123)	30 ± 9 (490)
	5 min	26 ± 7^#^	5 ± 1 [3–8]	30 ± 15 (80)	22 ± 10 (43)	32 ± 15 (33)	25 ± 12 (36)	21 ± 10 (15)	18 ± 7 (8)	15 ± 4 (49)	24 ± 10 (161)	34 ± 14 (142)	28 ± 8 (624)
CMP	1 min	68 ± 17	2 ± 1 [1–5]	41 ± 20 (41)	22 ± 13 (21)	54 ± 33 (3)	31 ± 13 (27)	30 ± 13 (9)	22 ± 11 (5)	21 ± 10 (33)	31 ± 17 (80)	39 ± 19 (94)	37 ± 18 (337)
	3 min	32 ± 10	3 ± 1 [1–7]	35 ± 18 (57)	24 ± 10 (25)	41 ± 44 (3)	27 ± 13 (29)	24 ± 12 (18)	27 ± 11 (13)	21 ± 12 (57)	26 ± 13 (120)	32 ± 16 (140)	30 ± 11 (514)
	5 min	23 ± 7	4 ± 1 [1–7]	34 ± 17 (56)	24 ± 10 (38)	33 ± 39 (5)	25 ± 11 (38)	22 ± 10 (22)	22 ± 12 (14)	19 ± 10 (72)	25 ± 11 (149)	31 ± 15 (159)	28 ± 9 (627)
WOP	1 min	76 ± 16^#^	2 ± 1 [1–4]	34 ± 16 (15)	35 ± 14 (22)	0 (0)	30 ± 17 (16)	30 ± 11 (12)	27 ± 20 (2)	19 ± 7 (12)	26 ± 10 (10)	34 ± 14 (28)	33 ± 10 (129)
	3 min	36 ± 7	4 ± 1 [2–6]	30 ± 17 (19)	29 ± 15 (28)	0 (0)	29 ± 17 (30)	28 ± 10 (20)	27 ± 11 (7)	22 ± 10 (28)	28 ± 14 (16)	32 ± 17 (37)	29 ± 9 (201)
	5 min	27 ± 5^#^	5 ± 2 [2–9]	29 ± 16 (29)	29 ± 15 (34)	19 ± 9 (2)	22 ± 10 (30)	24 ± 9 (22)	24 ± 12 (7)	18 ± 8 (23)	24 ± 10 (30)	31 ± 17 (39)	27 ± 9 (245)
COP	1 min	71 ± 14	2 ± 1 [1–4]	38 ± 21 (8)	25 ± 15 (13)	0 (0)	32 ± 19 (4)	33 ± 15 (16)	25 ± 13 (6)	27 ± 11 (22)	31 ± 15 (7)	41 ± 15 (4)	32 ± 12 (84)
	3 min	32 ± 7	4 ± 1 [2–6]	44 ± 17 (14)	23 ± 12 (18)	0 (0)	26 ± 15 (9)	24 ± 12 (19)	25 ± 12 (7)	27 ± 10 (29)	36 (1)	32 ± 17 (2)	29 ± 11 (117)
	5 min	25 ± 6	5 ± 1 [2–8]	33 ± 18 (21)	23 ± 14 (11)	0 (0)	27 ± 15 (10)	26 ± 14 (25)	21 ± 13 (8)	22 ± 9 (42)	26 ± 9 (4)	37 ± 7 (3)	26 ± 7 (141)
Overall	1 min	67 ± 19	2 ± 1 [1–5]	39 ± 19 (108)	29 ± 15 (84)	48 ± 20 (21)	29 ± 14 (83)	29 ± 13 (47)	23 ± 11 (22)	21 ± 10 (94)	30 ± 15 (288)	42 ± 18 (313)	36 ± 15 (1150)
	3 min	31 ± 9	3 ± 1 [1–7]	34 ± 18 (156)	26 ± 13 (105)	35 ± 19 (32)	25 ± 14 (122)	25 ± 11 (75)	26 ± 10 (35)	20 ± 10 (163)	25 ± 12 (471)	35 ± 17 (421)	31 ± 9 (1739)
	5 min	23 ± 7	3 ± 1 [1–9]	32 ± 16 (186)	24 ± 12 (138)	34 ± 18 (41)	24 ± 11 (139)	24 ± 11 (84)	21 ± 11 (42)	18 ± 8 (201)	23 ± 10 (616)	34 ± 16 (484)	23 ± 7 (2155)

CDP: Central Defensive Player; WDP: Wide Defensive Player; CMP: Central Midfield Player; WOP: Wide Offensive Player; COP: Central Offensive Player. HIR: High-intensity running.

SP: ‘Support Play’, MTR/ES: ‘Move to Receive/Exploit Space’, OVL/UDL: ‘Overlap/Underlap’, RWB: ‘Run with Ball’, RIB/PEN: ‘Run in Behind/Penetrate’, BIB: ‘Break into Box’, CD/PRE: ‘Close Down/Press’, COV: ‘Covering’, RR: ‘Recovery Run’. ‘Push up Pitch’ and ‘Interception’ were excluded due to the small number of actions. Physical-tactical average distances are reported as mean ± SD (m) and numeral in parenthesis indicates the total number of physical-tactical actions performed.

* Less high-intensity running distance than other positions (*P* < 0.01).

# Greater high-intensity running distance than CMP (*P* < 0.05).

### Contextualised Peak Periods – Team Trends

**FIG. 1:**
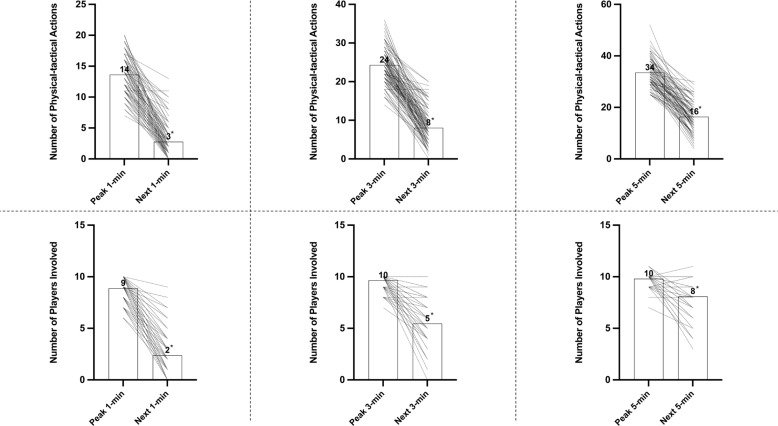
Team performance; number of physical-tactical actions and players involved during the peak and next 1-, 3-, 5-min periods. Numbers above the bars indicate mean values. Dotted lines indicate before-after values. *Difference from peak period (P<0.01).

During the peak 1-, 3- and 5-min periods, the teams covered 28–37% and 22–23% of the high-intensity distance (420 ± 82 m, 646 ± 125 m, and 842 ± 154 m, respectively), performing ‘Recovery Run’ and ‘Covering’ actions, respectively. However, they covered the largest proportion of their high intensity distance for ‘Support Play’ (12–13%) in possession. In the next 1-, 3- and 5-min periods, the teams had a deficit of 31%, 30%, and 17%, respectively, in high-intensity distance compared to the match average (ES: 0.5–0.8, *P* < 0.01). The teams covered 20–41% and 32–53% less high-intensity distance for ‘Covering’ and ‘Recovery Run’, respectively, compared to the match average (ES: 0.4–0.7, *P* < 0.01). Figure 1 shows the frequency of high-intensity actions and the numbers of players involved during the peak and next periods.

### Contextualised Peak Periods – Position-Specific Trends Central Defensive Player

**FIG. 2:**
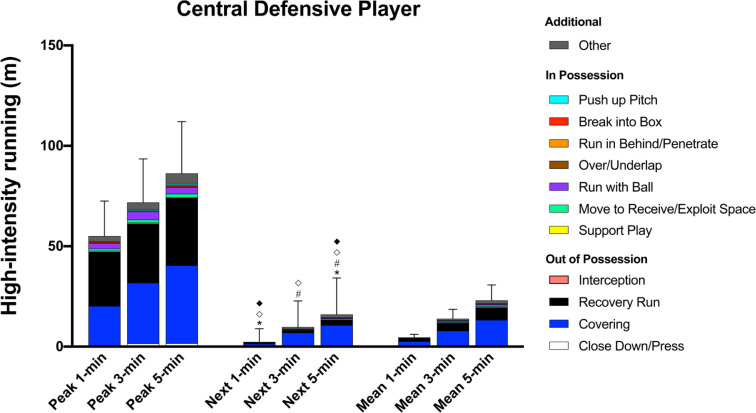
Central Defensive Player; contextualised distances at high-intensity in the peak 1-, 3-, and 5-min periods during the match, the subsequent period (next), and the match average (mean). *Difference from match average for ‘Covering’ (P<0.05). #Difference from match average for and ‘Recovery Run’ (P<0.01). ^⟡^Difference from match average for ‘Run in Behind/Penetrate’ (P<0.01). ^♦^Difference from match average for ‘Break into Box’ (P<0.01).

During the peak 1-, 3-, and 5-min periods, CDP performed ~80% of their high-intensity distance (55 ± 17 m, 72 ± 22 m, and 86 ± 26 m, respectively) out of possession whilst covering 39–49% of the distance for ‘Recovery Run’ and 36–45% for ‘Covering’ (Figure 2). CDP covered greater high-intensity ‘Covering’ distance than WOP and COP during all of the peak periods (ES: 0.7–1.4, P < 0.01) whilst also performing more ‘Recovery Run’ distance than COP (ES: 0.9–1.0, *P* < 0.01).

### Wide Defensive Player

**FIG. 3:**
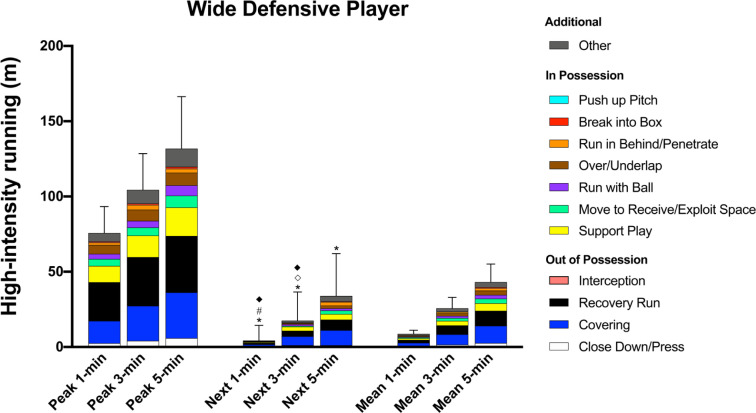
Wide Defensive Player; contextualised distances at high-intensity in the peak 1-, 3-, and 5-min periods during the match, the subsequent period (next), and the match average (mean). *Difference from match average for ‘Recovery Run’ (P<0.05). #Difference from match average for and ‘Support Play’ and ‘Run with Ball’ (P<0.05). ^⟡^Difference from match average for ‘Break into Box’ and ‘Run in Behind’ (P<0.01). ^♦^Difference from match average for ‘Move to Receive/Exploit Space’ (P<0.05).

During the peak 1-, 3-, and 5-min periods, WDP covered 28–34% and 20–23% of their high-intensity distance (76 ± 18 m, 104 ± 24 m, and 132 ± 35 m, respectively) for ‘Recovery Run’ and ‘Covering’, respectively whilst they covered 14% of the distance for ‘Support Play’, and 6–7% for ‘Over/Underlap’ (Figure 3). In possession WDP performed greater high-intensity ‘Support Play’ distance than CDP (ES: 0.7–1.0, *P* < 0.01) and ‘Over/Underlap’ than other positions (ES: 0.4–0.7, *P* < 0.01).

### Central Midfield Player

**FIG. 4:**
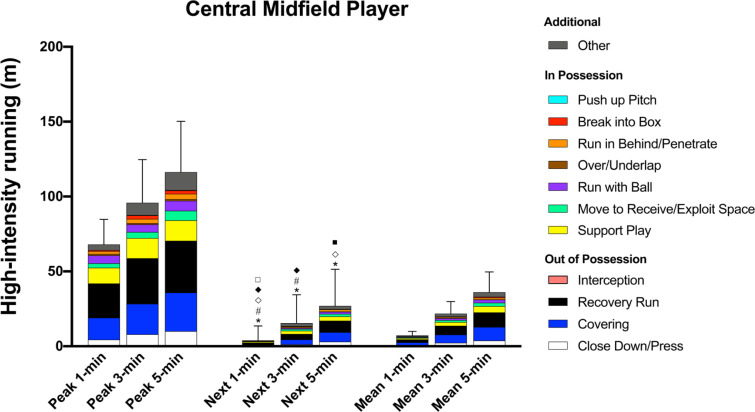
Central Midfield Player; contextualised distances at high-intensity in the peak 1-, 3-, and 5-min periods during the match, the subsequent period (next), and the match average (mean). *Difference from match average for ‘Covering’ (P<0.01). #Difference from match average for and ‘Close Down/Press’ (P<0.01). ^⟡^Difference from match average for ‘Break into Box’ (P<0.01). ^♦^Difference from match average for ‘Run with Ball’ (P<0.05). ^⟡^Difference from match average for ‘Move to Receive/Exploit Space’ (P<0.01). ^■^Difference from match average for ‘Support Play’ (P<0.05).

During the intensified 1-, 3-, and 5-min periods, CMP covered 29–33% and 21–22% of their high-intensity distance (68 ± 17 m, 96 ± 29 m, and 116 ± 34 m, respectively) for ‘Recovery Run’ and ‘Covering’, respectively, while they performed 12–15% of the distance for ‘Support Play’ (Figure 4). During all of the peak periods, CMP performed greater high-intensity ‘Recovery Run’ distance compared to COP (ES: 0.8–1.0, *P* < 0.01) whilst also covering greater ‘Support Play’ distance than CDP (ES: 0.7–0.8, *P* < 0.01).

### Wide Offensive Player

**FIG. 5:**
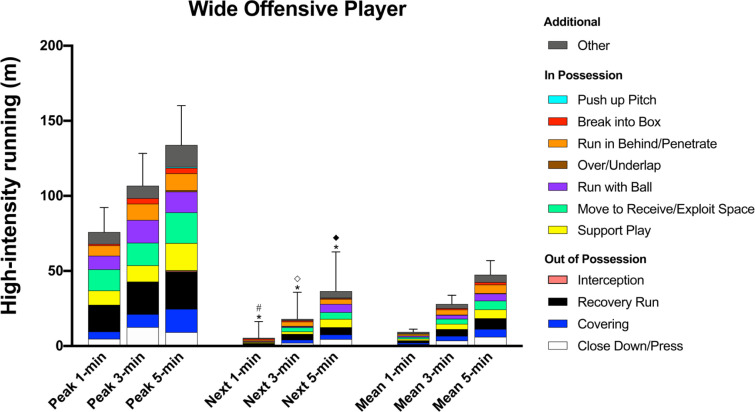
Wide Offensive Player; contextualised distances at high-intensity in the peak 1-, 3-, and 5-min periods during the match, the subsequent period (next), and the match average (mean). *Difference from match average for ‘Interception’ (P<0.01). #Difference from match average for and ‘Move to Receive/Exploit Space’ (P<0.01). ^⟡^Difference from match average for ‘Support Play’ (P<0.05) and ‘Run with Ball’ (P<0.01). ^♦^Difference from match average for ‘Run in Behind/Penetrate’ (P<0.05).

During the peak 1-, 3-, and 5-min periods WOP covered 19–23% of their high-intensity distance (76 ± 16 m, 107 ± 22 m, and 134 ± 26 m, respectively) for ‘Recovery Run’ whilst they performed 14–19% of their high-intensity distance for ‘Move to Receive/Exploit Space’, 10–14% for each ‘Support Play’ and ‘Run with Ball’, and 8–10% for ‘Run in Behind/Penetrate’ when in possession (Figure 5). During all of the peak periods WOP performed more high-intensity distances for ‘Run in Behind/Penetrate’ and ‘Move to Receive/Exploit Space’ than CDP, WDP, and CMP (ES: 0.5–1.6, *P* < 0.01) whilst also covering greater ‘Run with Ball’ distance than CDP and WDP (ES: 0.4–0.8, *P* < 0.05).

### Central Offensive Player

**FIG. 6:**
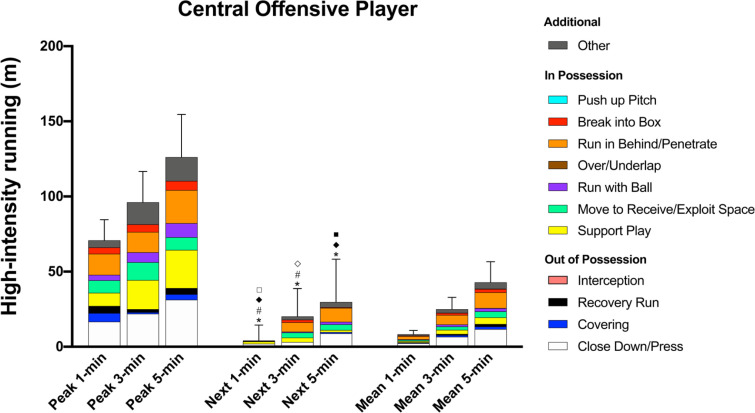
Central Offensive Player; contextualised distances at high-intensity in the peak 1-, 3-, and 5-min periods during the match, the subsequent period (next), and the match average (mean). *Difference from match average for ‘Recovery Run’ (P<0.01). #Difference from match average for and ‘Covering’ (P<0.01). ^⟡^Difference from match average for ‘Close Down/Press’ (P<0.05). ^♦^Difference from match average for ‘Break into Box’ (P<0.01). ^□^Difference from match average for ‘Run in Behind/Penetrate’, ‘Move to Receive/Exploit Space’, and ‘Run with Ball’ (P<0.01). ^■^Difference from match average for ‘Support Play’ (P<0.05).

During the peak 1-, 3-, and 5-min periods, COP covered 23–25% of their high-intensity distance (71 ± 14 m, 96 ± 21 m, and 126 ± 28 m, respectively) for ‘Close Down/Press’ whilst they ran 14–20% and 12–20% of the distance for ‘Run in Behind/Penetrate’ and ‘Support Play’, respectively (Figure 6). COP covered more high-intensity ‘Close Down/Press’ distance than other positions during all of the peak periods (ES: 0.4–2.5, *P* < 0.05). In possession, COP performed greater high-intensity ‘Run in Behind/Penetrate’ distance during all of the peak periods compared to other positions (ES: 0.4–2.2, *P* < 0.05) whilst also covering more distance for ‘Break into Box’ than CDP (ES: 0.7–0.9, *P* < 0.01) and WDP (ES: 0.6, *P* < 0.05).

## DISCUSSION

The present study is the first to consider the contextualised high-intensity distance covered during peak 1-, 3-, and 5-min periods of match-play and the following periods of each duration using a rolling average technique for individual and team performances. The contextualised data now provide important insights into how players/teams tactically perform in relation to high-intensity efforts during peak periods and how they altered their physical-tactical behaviour during the following periods. Nonetheless, some physical-tactical actions demonstrated inconsistency in different time durations of the next periods, and these physical-tactical data were position-specific. This may indicate that each position has certain physical-tactical actions to perform even after intensified periods of play, especially when tactically required to do so.

Numerous studies have examined match running performances in peak periods of play to provide an insight into intensified discrete periods (e.g., 1-min) [1, 3, 4, 11, 13, 18]. Supported by previous studies [2, 4, 11, 18], data demonstrates that CDP demonstrated the lowest locomotive demands whilst WDP and WOP exhibited the largest physical demands during intense periods. Additionally, the present study for the first time analysed the peak periods for team performances. Data indicate that during the peak 1-, 3-, and 5-min periods, almost all of the outfield players in different playing positions (9–10 players) were involved collectively covering high-intensity distances of ~400 m, ~650 m, and ~850 m, respectively. This indicates that all outfield players have to collectively perform some bouts of high-intensity actions during intensified periods of competition as a team. Nevertheless, such data provide only a rudimentary insight on physical performance.

The contextualised data revealed that out of possession ~20–35% of the high-intensity distance was covered by players for each ‘Recovery Run’ and ‘Covering’ whilst in possession ~10% was covered for ‘Support Play’. This may indicate that peak periods occur during a fast transition phase since such actions as ‘Recovery Run’ and ‘Support Play’ are commonly performed when the ball is quickly moved defensively or offensively during a quick transition [15]. This could also be supported with the team performance data where teams produced high-intensity ‘Recovery Run’ and ‘Support Play’ actions the most out of possession and in possession, respectively. This could be due to players/teams executing more high-intensity actions during decisive phases of play than normal situations [30]. Nevertheless, since the present study did not analyse phases of play (e.g., attack-to-defence transition phases), it is difficult to fully conclude whether intensified periods take place during fast transition phases or not. Thus, future studies should attempt to condense contextualised actions into the phases of play to provide additional granularity.

However, the contextualised data during the peak periods were position-specific. For instance, the key high-intensity tactical actions during the peak periods for CDP were ‘Covering’ and ‘Recovery Run’. This is possibly due to one of their main defensive duties, which is to defend the space left behind particularly when a turnover in possession occurs [31]. In addition to these, ‘Support Play’ was another main physical-tactical action for WDP and CMP, but there was a bespoke action for WDP (‘Over/Underlap’). This clearly demonstrates their attacking responsibilities during the peak periods. For instance, WDP and CMP should perform ‘Support Play’ to become involved in the attacking/defence-to-attack transition phase to produce a promising attacking threat [7, 15]. Furthermore, the key high-intensity tactical activities for WOP were ‘Move to Receive/Exploit Space’, ‘Run in Behind/Penetrate’, and ‘Support Play’, and ‘Run with Ball’ when in possession and ‘Recovery Run’ when out of possession. By contrast, ‘Close Down/Press’, ‘Run in Behind/Penetrate’, and ‘Support Play’ were the key high-intensity tactical activities for COP. The data clearly exhibit their specific tactical roles during intensified periods. For example, COP should aggressively close down/press the opponent to make it hard for them to advance their attacking play or regain possession when out of possession [32] whilst they should also perform attacking actions (e.g., ‘Run in Behind/Penetrate’) to create promising chances when in possession [7]. Such position-specific tasks could be used to replicate intensified periods during training matches as the 11v11 training matches could offer the players resources to train the most demanding episodes of match-play [6]; however, it should be acknowledged that it is unlikely to provide the necessary ‘overload’ desired at times. Additionally, these position-specific trends could be easily translated into training sessions using the average distance per physical-tactical action and average number of actions during the peak periods (Table 2). For example, whilst CMP is driving through the middle running with the ball at high-intensity (~20 m), WDP could perform a high-intensity over/underlapping action (~35 m) in a wide area from the middle to the final third. Once CMP pass the ball to WDP, they could produce a ‘Break into Box’ action (~20 m) and WDP could cross the ball at the end of the action. Both then produce ‘Recovery Run’ actions (~30–40 m) to get goal side of the ball to replicate fast transition phases of the peak periods. Although this type of work has been previously attempted to replicate player’s physical demands whilst simultaneously reflecting position-specific game situations [5, 14], they used average physical demands, which may underestimate the true match demands [9]. This could also be supported by the fact that the average distance per action performed during intensified periods is greater (e.g., ~35 m during the peak 1-min period, Table 2) than that performed during the entire match (~20 m) [7]. Thus, these contextualised peak distance data could help practitioners better prescribe not only position- but player-specific drills with the true peak demands.

Decrements that follow after the peak periods have been previously examined alongside analysing intensified periods of play [1–3, 18]. However, most studies used a predefined technique (e.g., 0–5, 5–10 min etc.), which can under or overestimate the true physical demands of the peak period and the subsequent period, respectively [28]. Moreover, different speed thresholds for high-intensity running during the peak periods have been used (> 14.0–19.8 km · h^−1^), which makes it difficult to compare across studies. Despite these short-comings, the general consensus from previous research was that the transient decrements in high-intensity running occur after intense periods, which agrees with the findings of the present study. Additionally, the present study analysed shorter durations of the next periods to evaluate more detailed temporal changes after the peak periods of match-play. Data demonstrated that players experienced more pronounced reductions in high-intensity running in the next 1-min period (~50%) compared to the next 3- and 5-min period (~25–30%) after the peak periods of play. This prominent short-term fluctuation during the next 1-min period seems likely due to less energy available from creatine phosphate hydrolysis since creatine phosphate concentrations could be significantly diminished after some bouts of high-intensity actions [33]. That said, similar trends for team performances were observed during the subsequent periods to individual performances. This might indicate that teams briefly modify their collective tactical behaviour after intensified periods during matches, which could also be supported by the number of high-intensity actions and players involved in the following periods (Figure 1). Yet, it could be due to reduced playing time (e.g., ball out of play); thus, it is difficult to fully determine without context whether transient decrements are down to fatigue or tactical alterations/pacing strategies or reduced playing opportunities.

The present study for the first time provides important insights on how players/teams alter their tactical behaviour during the next 1-, 3-, and 5-min periods through integrating the physical-tactical metrics. Players/teams changed their tactical performances by covering ~20–55% less high-intensity distances for ‘Covering’ and ‘Recovery Run’ during all of the subsequent periods compared to the match average. Since both players and teams consistently ran less high-intensity distance for such variables, this may denote that they tend to be defensively in a good tactical position/formation when out of possession after intense periods whilst covering less high-intensity distance. This could be due to the defensive phases of play being more physically taxing [34]. In possession, players performed ~30–90% less ‘Run with Ball’ distances in the next periods compared to the match average, which may be due to such actions (e.g., dribbling) causing an increased energy cost when compared to running without the ball [35] or due to player time running with the ball being small [36]. However, even with such context it is still challenging to fully explain why transient decrements occur in subsequent periods. Thus, the systematic use of video to check each player and to add more layers of information together with effective playing time (e.g., only in-play time) may provide clearer insights into how players/teams alter their physical-tactical actions after intense periods [34].

Data demonstrates that physical-tactical trends during the subsequent periods were also position-specific. For instance, WDP covered ~30–50% less high-intensity distance for ‘Recovery Run’ in all of the next periods compared to the match average. ‘Recovery Run’ is when players run back toward own goal to get goal side of the ball when out of position [22], therefore this might specify that WDP modulates their physical-tactical performances by being less involved in the attacking/transition phase during the next periods. That said, it would be of greater interest if measuring the ability of the player to be involved in the subsequent attack after the tactical modulation to evaluate the effectiveness of the player. However, certain physical-tactical actions exhibited inconsistency in different time durations of the next periods. For instance, COP covered ~80–100% less high-intensity distances for ‘Break into Box’ in the next 1- and 5-min periods compared to the match average; however, they covered ~20% more distances for this action during the next 3-min period. This may indicate that players selectively produce high-intensity running particularly when tactically required to do so. Yet, it is still difficult to draw conclusions since performances are influenced by context (e.g., no need to perform or choosing not to perform physical-tactical actions). Thus, more context should be provided to better understand ‘WHY’ players perform less physical-tactical actions during the subsequent period after the peak passage.

### Limitation

Firstly, the present study did not quantify acceleration/deceleration efforts during matches. Although most of these efforts do not reach high-intensity speed thresholds, they are very frequent during matches and are extremely taxing mechanically [37]. Thus, these actions should be incorporated and contextualised to have a comprehensive understanding of the true physical demands with tactical purposes. Moreover, although contextualised data include high-intensity running activities with a single (~75%) and hybrid tactical (~15%) actions with unclassified movements as ‘Other’ (~10%), the present study combined the singular actions and the primary action of the hybrid actions to simplify data output. Thus, future research should evaluate hybrid actions to provide more transparency and insight to practitioners. Furthermore, since the present study analysed general positional data (e.g., WDP and CMP) and these were derived from different formations (e.g., 4-3-3, 3-5-2, etc.), this could have impacted data due to formations/player playing style influencing match performance [38, 39]. Therefore, it is warranted to evaluate the effects of formation or different playing styles of players (e.g., central defensive or attacking midfielders) on match physical-tactical performances.

## CONCLUSIONS

The contextualised distance data could help coaches and/or practitioners better prescribe position- and player-specific training drills, and may help improve the understanding of transient decrements in high-intensity running during the subsequent periods. However, since it is still complex due to numerous influencing factors, additional context should be provided to have a better understanding of the transient decrements in high-intensity running.

## Conflict of interest

The authors have no conflict of interest to declare.
